# Development and initial validation of the Generalized Tracking Questionnaire

**DOI:** 10.1371/journal.pone.0234393

**Published:** 2020-06-11

**Authors:** Francisco J. Ruiz, María B. García-Martín, Juan C. Suárez-Falcón, Luna Bedoya-Valderrama, Miguel A. Segura-Vargas, Andrés Peña-Vargas, Ángela M. Henao, Jorge E. Ávila-Campos

**Affiliations:** 1 Faculty of Psychology, Fundación Universitaria Konrad Lorenz, Bogotá, Colombia; 2 Department of Methodology of the Behavioral Sciences, Faculty of Psychology, Universidad Nacional de Educación a Distancia (UNED), Madrid, Spain; Sapienza, University of Rome, ITALY

## Abstract

The concept of rule-governed behavior (RGB) has been used in the behavior-analytic literature as a way to analyze complex human behavior, including thinking and problem-solving. Relational frame theory suggests the existence of two main functional types of RGB termed pliance and tracking. In this paper, we describe the development of the Generalized Tracking Questionnaire (GTQ) and the preliminary evaluation of its psychometric properties and validity through three studies, with a total of 1155 participants. In Study 1, a pool of items describing the main characteristics of generalized tracking was designed and evaluated by experts on the RFT account of RGB. The resulting 11 items were administered to 460 undergraduates to examine the understandability and psychometric quality of the items. The exploratory factor analysis indicated that the GTQ can be seen as a unidimensional scale, with all items exhibiting high factor loadings and corrected item-total correlations. In Study 2, the GTQ was administered online to a sample of 464 non-clinical participants and a clinical sample of 125 participants. The one-factor model of the GTQ obtained a good fit in the conducted confirmatory factor analysis. The GTQ showed measurement invariance across gender and clinical and nonclinical participants. It also obtained excellent internal consistency and correlated in theoretically coherent ways with other constructs. In Study 3, the GTQ and a neuropsychological battery of executive functions were administered to 105 participants. The GTQ showed statistically significant, medium-size correlations with working memory tests, verbal fluency, planning, and behavioral inhibition. In conclusion, the GTQ seems to be a promising measure to advance in the empirical analysis of functional classes of RGB.

## Introduction

The understanding and explanation of complex human behavior is the cornerstone of all paradigms in psychology. Within behavior analysis, Skinner coined the term rule-governed behavior to advance towards the explanation of thinking and problem-solving [1,2]. In the following decades, theoretical and empirical analysis of rule-governed behavior became one of the main research lines in behavior analysis and in its modern derivations such as contextual behavioral science [3,4]. Accounts in terms of rule-governed behavior have been developed to explain, among others, psychopathology [5–8], psychological therapy [9,10], decision making [11], executive functions [12], moral behavior [13], behavioral anthropology [14], or behavioral pharmacology [15]. Across these domains, a central topic of research in rule-governed behavior has been the differential outcomes of rule-governed behavior vs. contingency-shaped behavior.

Contingency-shaped behavior is behavior directly controlled by its consequences and is a type of learning seen in human and nonhuman animals [1,16]. For instance, a rat might learn to press the lever of the Skinner’s box only after one minute since the last access to the reinforcer (e.g., food) because emitting the behavior before is not associated with reinforcement. Rule-governed behavior, however, is exclusive of human beings and is behavior that is controlled by antecedent verbal stimuli provided by another person or by the own individual [17], who can act as the speaker and listener within the same skin [18]. Following the example above, rule-governed behavior would consist of the individual following an experimenter rule (e.g., “You will obtain money by pressing the key ‘P’ after one minute of delay”) or his/her own derived rule (e.g., “I win money by pressing the key ‘P’ after some time”).

Vast empirical research has shown that contingency-shaped behavior in nonhuman animals is sensitive to changes in reinforcement schedules (i.e., after some experience, the rat will behave according to the newly arranged contingencies) [19]. However, human operant research soon showed intriguing findings: whereas preverbal children showed performances coherent with those seen in experiments with nonhuman animals [20,21], verbal human participants performed considerably different [22]. This difference in performance was soon attributed to the interference provoked by participants’ self-talk during the experiments in the form of rules [23]. Accordingly, researchers began to compare human behavior under the control of instructions (i.e., rule-governed behavior) provided by the experimenter with contingency-shaped behavior (i.e., participants were not instructed; they learned during the experiment how to obtain reinforcement by trial and error). The findings of this research line showed that participants who are instructed tend to show more insensitivity to changes in contingencies (e.g., changes in reinforcement schedules) than participants who were shaped by them [3]. This phenomenon was called *insensitivity to contingencies* [3,24–25].

Zettle and Hayes attempted to provide a classification of functional classes of rule-following useful to explain the insensitivity to contingencies phenomenon [10]. This account was subsequently incorporated in relational frame theory [26], which provides a functional-analytic explanation of the core characteristics and behavioral processes involved in rule-governed behavior, including the conceptualization of verbal stimuli, the generation, meaning and understanding of rules, and rule-following [17,27]. For the sake of brevity, in this article, we will only focus on rule-generation and rule-following.

According to Zettle and Hayes, the most fundamental functional classes of rule-following are pliance and tracking [10]. On the one hand, pliance is rule-following due to a history of multiple exemplars in which the speaker provided the listener with reinforcement contingent on the correspondence between the rule content and the listener’s behavior [5,10,27]. On the other hand, tracking is rule-following due to a history of multiple exemplars in which the correspondence between the rule content and the listener’s behavior is reinforced by the natural consequences that are derived from the way the world is arranged [5,28]. The main difference between pliance and tracking is the *apparent* source of reinforcement for rule-following: social or arbitrary in the case of pliance and nonarbitrary in the case of tracking (i.e., the displayed behavior causes the consequence). The word *apparent* is relevant here to emphasize that rules are antecedent events and that the consequences contacted when rule-following only affect the future value of the rules. In this sense, the present value of the rules is determined by the history of the listener [17], which makes it very difficult to induce pliance and tracking rule-following in experimental settings [29].

Pliance is the first functional class of rule-following developed because of its relational simplicity [13]. The development of tracking is produced after some experience with pliance. This ontogenetic origin of rule-governed behavior might explain the phenomenon of *insensitivity to contingencies*. A learning history heavily based on pliance, or an experimental context that actualizes pliance functions, will lead the participant to follow the rule provided by the experimenter because of its antecedent verbal functions (e.g., “She said that I have to press the “P” key repeatedly” or “If I don’t press the “P” key, she might get disappointed”) without contacting the change in the experimental contingencies. Specifically, insensitivity to contingencies will be more likely if children are not exposed to interactions that help them verbally contact the natural consequences of their behaviors (i.e., tracking). If this occurs, pliance will probably generalize to the extent to generate a pattern of rule-following–generalized pliance–characterized for having social approval as the main source of reinforcement [5,7,30].

The link between generalized pliance and insensitivity to contingencies has been tested recently thanks to the development of the Generalized Pliance Questionnaire and the Generalized Pliance Questionnaire–Children [31,32]. The GPQ has shown strong correlations with the performance on contingency-shifting tasks [33] such as the Wisconsin Card Sorting Task [34,35] and the Iowa Gambling Task [36,37]. Generalized pliance has also been associated with psychopathology because it increases the likelihood of the individual losing contact with relevant sources of positive reinforcement due to the insensitivity to contingencies effect [5,7]. Indeed, generalized pliance is part of the psychological inflexibility model of psychopathology advocated by acceptance and commitment therapy (ACT) [38]. Empirical evidence with the GPQ supports the potential maladaptive role of generalized pliance given its positive correlations with emotional symptoms, psychological inflexibility, repetitive negative thinking, dysfunctional attitudes, obstruction in valued living, and negative correlations with life satisfaction and mindfulness.

Tracking is more likely to be sensitive to direct contingencies because rule-following is due to the apparent causal relationship between the actual behavior and the consequences contacted [7,10,28]. According to this, a change in the relationship between behavior and contingencies might lead the individual to modify his or her behavior. Furthermore, when an individual who has strong relational skills and has also been exposed to multiple interactions in which he or she has been guided to observe and describe functional relationships among events, a pattern of rule-following that we call *generalized tracking* will be developed. Accordingly, generalized tracking involves the motivation and skill to establish functional relationships among behaviors and their consequences and to adjust behavior according to them. Note that generalized tracking involves the individual behaving both as speaker and listener.

To our knowledge, the term *generalized tracking* has not been used in behavior analysis or contextual behavioral literature, although mentioning tracking as a skill has been frequent [5,30,39]. For instance, influential authors have provided a second definition of tracking that coincides with the conceptualization of generalized tracking: “observing and describing functional relationships among psychological events (e.g., noticing the consequence of a behavior; drawing out rules based on observation) that could then function as tracks in the first sense. For example, modifying a recipe after having used new ingredients that made the cake even more delicious” [39]. To avoid confusion with the two definitions of tracking, we prefer to use the term *generalized tracking* when referring to this pattern of rule-governed behavior.

Generalized tracking might be seen as the most flexible rule-governed behavior. In this sense, an RFT conceptualization of executive functions as a subset of rule-governed behavior characterized by rule flexibility has been provided [12]. Accordingly, the conceptualization and empirical analysis of generalized tracking might provide an advance towards the functional analysis of executive functions. These are a set of interrelated cognitive processes involved in complex activities directed towards concrete objectives [40]. Current conceptualizations of executive functions highlight three control mechanisms: working memory, inhibitory control, and cognitive flexibility [41]. Working memory consists of holding new information for brief periods and establishing specific objectives according to the situation requirements [42]. Inhibitory control refers to the suppression of predominant, but irrelevant, responses to progress towards an objective [43]. Lastly, cognitive inflexibility refers to the tendency to maintain current behavior disregarding negative feedback [44].

Contrary to generalized pliance, to our knowledge, there are not self-reports of generalized tracking available. Accordingly, the current study aimed to develop a measure of generalized tracking–the Generalized Tracking Questionnaire (GTQ)–for adults from the general nonclinical and clinical populations. In so doing, we conducted three studies, with a total of 1155 participants. Study 1 aimed to develop items describing the main characteristics of generalized tracking and to preliminarily analyze the understandability and psychometric quality of the items by conducting an exploratory factor analysis. In Study 2, we conducted a confirmatory factor analysis of the factor structure found for the GTQ in the previous study and measurement invariance analyses across gender and clinical and nonclinical participants. Also, the correlations between the GTQ and other self-reports were analyzed. Lastly, in Study 3, we explored the correlations of the GTQ with a neuropsychological battery of executive functions.

## Study 1: Item development and preliminary analysis of factor structure and internal consistency

### Materials and methods

The procedures followed in the research reported in the manuscript were approved by the Bioethics Committee of Fundación Universitaria Konrad Lorenz. Written informed consent was obtained in all studies reported in the manuscript. The dataset used in this study can be obtained at https://osf.io/r2gb4/.

#### Item development

An initial pool of items reflecting generalized tracking was generated on a group basis. The group was led by the first and seventh authors and consisted of students of different levels interested in RFT research. The concepts of RGB, pliance, and tracking were discussed in four 2-hour sessions. Then, generalized tracking was defined to the participants and items were designed collaboratively. The definition of generalized tracking indicated that: “Tracking is a functional class of rule-following in which behaving as stated in the rule was reinforced by the natural consequences of the action. Generalized tracking involves the following interrelated characteristics: (a) the individual’s skill to discriminate the changes in the context and adjust his/her behavior consequently; (b) the individual’s skill to adjust his/her behavior according to the natural consequences of his/her actions; and (c) the individual’s skill to allow his/her thinking to be shaped by how things work. Participants were not asked to design items containing negative statements that would serve as reverse-scored items because they usually generate problems when conducting factor analyses [45,46].

Approximately 25 items were generated following the definition of generalized tracking. After discussing the adequacy of the items, 11 items were retained. This initial pool of 11 items was given to three experts in RFT and RGB with Ph.D. degrees. The experts suggested slight modifications in phraseology and approved the pool of 11 items (see S1 and S2 Tables). A 7-point, Likert-type scale (7 = *always*; 1 = *never true*) was adopted for the GTQ. This initial version of the GTQ was then administered to a sample of undergraduates to analyze its psychometric properties preliminarily.

#### Participants

*Sample 1*. The sample used in this study consisted of 460 undergraduates (age range 18–41, *M =* 20.90, *SD =* 2.73, 66.5% females) from a Colombian university. Most of them were studying Psychology (94.6%).

#### Procedure

Participants were approached at the beginning of a regular class where they were invited to participate. Individuals who agreed to do so signed the informed consent and were given the GTQ. Participants were asked to report if they had problems understanding some items. After completing the study, the aims of the research were debriefed to the participants and they were also thanked for their participation. No incentives were provided.

### Results and conclusions

Participants did not show problems understanding the GTQ items. The missing data were imputed using the matching response pattern of PRELIS-LISREL© (version 8.71) [47], which is suitable for ordinal variables [48]. In this imputation method, the value to be substituted for the missing value of a single case is obtained from another case (or cases) having a similar response pattern over the remaining items of the test. Only three values were missing, which represents 0.06% of the data.

The software Factor 10.9.02 [49] was used to conduct an exploratory factor analysis (EFA). Given the lack of multivariate normality in the data (multivariate Mardias’ test of skewness and kurtosis = 414.26, p < .001), we selected the robust maximum likelihood (MLR) estimation method with Pearson’s product-moment correlation matrix and the asymptotic variance-covariance matrix. The number of factors to retain from the EFA was determined using the optimal implementation of parallel analysis based on minimum rank factor analysis (PA) [50].

Bartlett’s statistic was statistically significant (1561.9(55), *p* < .001), and the result of the Kaiser-Meyer-Olkin (KMO) test was good (.88). The parallel analysis suggested extracting only one factor that accounted for 48.9% of the variance. Table 1 shows that factor loadings were adequate for all items: from .45 (Item 5) to .72 (Item 8).

**Table 1:** Initial pool of items of the GTQ, factor loadings, and corrected item-total correlations.

Items	Factor loading	Corrected item-total correlation
1. Cuando veo que algo no está funcionando, intento algo diferente [When I see that something is not working, I try something different]	.64	.59
2. Disfruto descubriendo cómo funcionan las cosas y llegando a mis propias conclusiones [I enjoy finding out how things work and reaching my own conclusions]	.54	.51
3. Me adapto fácilmente a los cambios [I adapt easily to changes]	.53	.48
4. Tengo facilidad para encontrar soluciones novedosas a los problemas [I am able to find novel solutions to problems]	.62	.57
5. Tomo decisiones basándome en mi experiencia y no en lo que los demás dicen [I make decisions based on my experience and not on what others say]	.45	.42
6. Me gusta probar distintas maneras de hacer las cosas para ver cuál es mejor [I like to try different ways of doing things to see which is better]	.65	.59
7. Soy bueno encontrando formas más efectivas de realizar tareas [I'm good at finding more effective ways to perform tasks]	.64	.58
8. Si noto que algo no funciona, cambio mi forma de actuar rápidamente [If I notice that something is not working, I change my way of acting quickly]	.72	.65
9. Aprendo de las consecuencias de mis acciones con facilidad [I learn from the consequences of my actions with ease]	.57	.53
10. Cuando me doy cuenta de que estoy equivocado, cambio mi forma de pensar y actuar [When I realize that I am wrong, I change my way of thinking and acting]	.48	.44
11. Tomo decisiones basándome en los resultados que he obtenido anteriormente [I make decisions based on the results I have obtained previously]	.61	.58

An assessment of unidimensionality was conducted by computing the Unidimensional Congruence (UniCo), Explained Common Variance (ECV), and Mean of Item Residual Absolute Loadings (MIREAL) indexes. Values larger than .95 and .85 in UniCo and ECV, respectively, suggest that data can be treated as essentially unidimensional, whereas for the MIREAL, a value lower than .30 suggests unidimensionality [51]. The values of UniCo (.97) and MIREAL (.22) suggested that the GTQ can be treated as a unidimensional measure, whereas the value of ECV (.83) approached this conclusion.

In summary, the results of the conducted EFA suggested that the GTQ can be treated as a unidimensional measure. All items showed good factor loadings. Accordingly, we computed the corrected item-total correlations with SPSS 25^©^ to analyze the discrimination item index in the one-factor model. Table 1 also shows that all items showed good discrimination with corrected item-total correlations higher than .30 and ranging from .42 (Item 5) to .65 (Item 8).

Cronbach’s alpha and McDonald’s omega coefficients were computed providing percentile bootstrap 95% confidence intervals (CI) with the MBESS package in R [52,53]. The alpha coefficient of the GTQ was .85 (95% CI [.83, .87]), whereas the omega coefficient was also .85 (95% CI [.83, .87]). According to the results of Study 1, we decided to retain the 11 items of the GTQ.

## Study 2: Confirmatory factor analysis, measurement invariance testing and convergent construct validity

This study conducted a confirmatory factor analysis in Samples 2 and 3 of the one-factor structure of the GTQ found in Study 1 with Sample 1. Measurement invariance analyses were conducted for gender and type of sample (clinical vs. nonclinical samples). Additionally, the correlations between the GTQ and other self-report measures in these samples were computed. We expected that the GTQ would correlate negatively with generalized pliance, emotional symptoms and dysfunctional coping strategies such as experiential avoidance, cognitive fusion or repetitive negative thinking. Also, generalized tracking should show positive correlations with measures of progress in values, life satisfaction, and self-efficacy.

### Materials and methods

The procedures followed in the research reported in the manuscript were approved by the Bioethics Committee of Fundación Universitaria Konrad Lorenz. Written informed consent was obtained in all studies reported in the manuscript. The dataset used in this study can be obtained at https://osf.io/r2gb4/.

#### Participants

*Sample 2*.This sample consisted of 464 participants (67.0% females) with an age range between 18 and 67 years (*M* = 26.80, *SD* = 9.53). All the participants were Colombian and they responded to an anonymous internet survey distributed through social media.

*Sample 3*. This sample consisted of 125 participants (76.8% females) with an age range between 18 and 56 years (*M* = 28.63, *SD* = 7.82). All the participants showed interest in participating in a clinical study analyzing the efficacy of a brief psychological intervention for depression and generalized anxiety disorder.

#### Instruments

*Generalized Pliance Questionnaire– 9 (GPQ-9) [31].* The GPQ-9 is the abbreviated form of the GPQ, which consists of 18 items. The GPQ was designed to measure generalized pliance and it is graded on a 7-point Likert-type scale (7 = *always*; 1 = *never true*). We expected that the GTQ would show moderate negative correlations with the GPQ-9. The GPQ-9 showed alphas of .92 and .93 for Samples 2 and 3, respectively.

*Acceptance and Action Questionnaire–II (AAQ-II) [54].* The AAQ-II consists of 7 items that are graded on a 7-point Likert-type scale (7 = *always true*; 1 = *never true*) that measures experiential avoidance. The Spanish version has proven to have good psychometric properties and a one-factor structure in Colombian samples [55]. The AAQ-II was administered because a generalized tracking measure might negatively correlate with experiential avoidance scores. The AAQ-II showed alphas of .92 and .88 for Samples 2 and 3, respectively.

*Depression Anxiety and Stress Scales– 21 (DASS-21) [56,57].* The DASS-21 is an instrument conformed by a 21-item with a 4-point Likert-type scale (3 = *applied to me very much or most of the time*; 0 = *did not apply to me at all*) that measures negative emotional states experienced during the last week. The DASS-21 has a hierarchical factor structure with three first-order factors (Depression, Anxiety, and Stress) and a second-order factor that is an overall indicator of emotional symptoms. The DASS-21 was administered because a measure of generalized tracking might show negative correlations with emotional symptoms. The DASS-21 showed alphas of .95 and .93 for the total scale for Samples 2 and 3, respectively. Concerning the DASS-21 subscales, Depression showed alphas of .92 and .89, Anxiety of .87 and .86, and Stress of .88 and .84.

*General Self-Efficacy Scale (GSES) [58,59].* The GSES is a 10-item, 4-point Likert scale (4 = completely true; 1 = not at all true) that aims to measure people’s belief about their ability to cope with a wide range of stressors. It has good internal consistency and a one-factor solution. The GSES was administered because a measure of generalized tracking might show positive correlations with general self-efficacy. The GSES showed an alpha of .89 in Sample 2.

*Cognitive Fusion Questionnaire (CFQ) [60,61].* The CFQ is a questionnaire that through a 7-item, 7-point Likert-type scale (7 = *always*; 1 = *never true*) measures cognitive fusion as averaged across contexts. Together with the AAQ-II, it is one of the most frequently used measures of ACT processes. In this study, the CFQ obtained an alpha of .91 in Sample 3.

*Valuing Questionnaire (VQ) [62].* The VQ is a questionnaire that through a 10-item, 7-point Likert (6 = *completely true*; 0 = *not at all true*) self-report instrument assesses valued living averaged across life areas during the last week. It comprises two subscales: Progress and Obstruction. In this study, the VQ obtained alphas of .82 and .74 for Progress and Obstruction, respectively, in Sample 3.

*Perseverative Thinking Questionnaire (PTQ) [63].* The PTQ is a 15-item, 5-point Likert (4 = *almost always*; 0 = *never*) self-report instrument. It is a content-independent self-report of repetitive negative thinking in response to negative events. In this study, the PTQ obtained alphas of .97 and .96 for Samples 2 and 3, respectively.

*Satisfaction with Life Survey (SWLS) [64,65].* This survey consists of a 5-item, graded with a 7-point Likert-type scale (7 = *strongly agree*; 1 = *strongly disagree*) that measures self-perceived well-being. The SWLS has proven to have good psychometric properties, convergent validity, and a one-factor structure in Colombia [66]. In this study, the alpha of the SWLS was .90 in Sample 2.

#### Procedure

Participants in Sample 2 responded to an anonymous internet survey distributed through the Internet and social media. The survey was called “Survey of Emotional Health in Colombia” and was completed on the platform www.typeform.com. After finishing data collection, a general report was sent to the participants who provided an email address for that purpose. Afterward, personal scores and options for receiving low-cost psychological treatment were provided when requested by the person. No incentives were provided for participation.

Participants in Sample 3 showed interest in participating in a clinical trial. In the filtering process, they responded to an online survey on the platform www.typeform.com. Afterward, participants were contacted to conduct a clinical interview to assess if they met the inclusion criteria for the clinical trial.

All participants provided informed consent and were given a questionnaire packet. Participants in Sample 2 responded to the GTQ, GPQ-9, AAQ-II, PTQ, DASS-21, SWLS, and GSES. Participants in Sample 3 responded to the GTQ, GPQ-9, AAQ-II, CFQ, PTQ, DASS-21, and VQ.

#### Results and conclusions

Before conducting the statistical analyses, data were examined searching for missing values. In Sample 2, the only missing data were of one participant with missing values in all the GTQ items. Thus, the participant’s data were deleted. There were no missing data in Sample 3.

*Validity evidence based on internal structure*. As the EFA conducted in Study 1 indicated that the GTQ seems to be a unidimensional measure, confirmatory factor analyses (CFA) were conducted with Samples 2 and 3 merged to analyze the fit of a one-factor model. Given the lack of multivariate normality in the data (multivariate Mardias’ test of skewness and kurtosis = 977.32; p < .001), the MLR estimation method with covariance matrix and the asymptotic variance-covariance matrix was adopted to conduct the CFA using LISREL^©^. We computed the Satorra-Bentler chi-square test and the following goodness-of-fit indexes for the one-factor model: (a) the root mean square error of approximation (RMSEA); (b) the comparative fit index (CFI); (c) the non-normed fit index (NNFI); and (d) the standardized root mean square residual (SRMR). According to Hu and Bentler [67], RMSEA and SRMR values of .08 represent a good fit, and values below .05 represent a very good fit to the data. Concerning the CFI and NNFI, values above .90 indicate well-fitting models, and above .95 represent a very good fit to the data.

**Fig 1:**
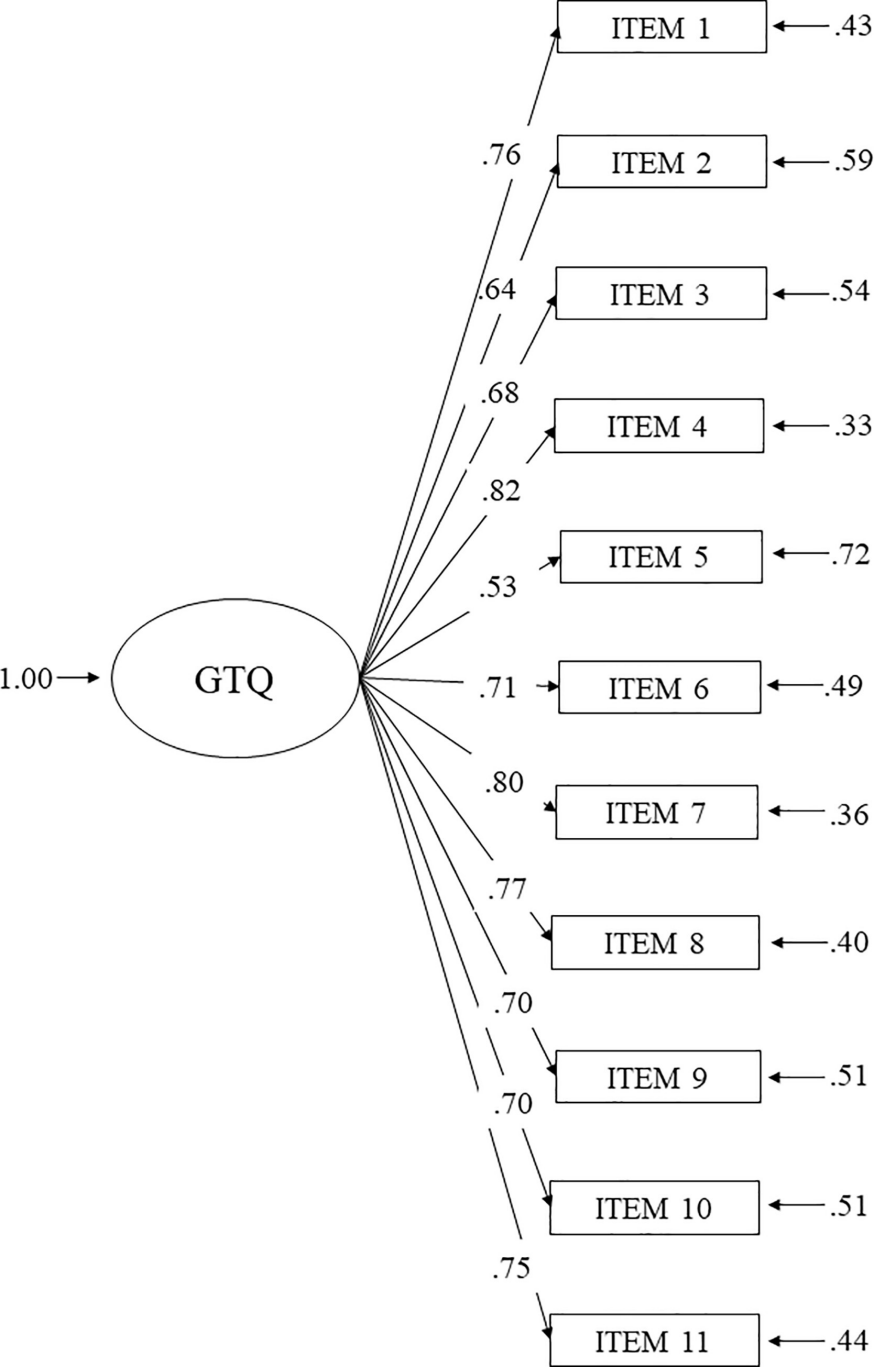
Completely standardized solution of the GTQ one-factor model conducted with Samples 2 and 3.

The overall fit of the one-factor model in the GTQ was good: χ^2^(44) = 200.94, *p* < .01; RMSEA = 0.078, 90% CI [0.067, .089], CFI = .98, NNFI = .98, SRMR = 0.047. Fig 1 depicts the results of the completely standardized solution of the one-factor model. Modification indices recommended allowing error terms between items 10 and 11. When doing so, the fit of the GTQ improved (χ^2^ (43) = 154.58, p < .01; RMSEA = 0.066. 90% CI [0.055, 0.078], NNFI = .98, CFI = .99, SRMR = 0.043).

*Measurement invariance across gender and sample*. Metric and scalar invariance across gender and samples (i.e., nonclinical vs. clinical samples) were conducted following the guidelines provided by Jöreskog [48] and Millsap and Yun-Tein [68]. In so doing, the relative fits of three increasingly restrictive models were compared: the multiple-group baseline model, the metric invariance model, and the scalar invariance model. The multiple-group baseline model allowed the eleven unstandardized factor loadings to vary across groups while the factor structure (the number of factors and pattern of item-factor loadings) is identical across groups (i.e., configural invariance). The metric invariance model was nested within the multiple-group baseline model and placed equality constraints on those loadings across groups (i.e., weak invariance model). Lastly, the scalar invariance model was nested within the metric invariance model and tested whether the factor loadings and the item intercepts were the same across groups (i.e., strong invariance model). The models were compared taking into account the differences in RMSEA, CFI, and NNFI indexes between nested models. The more constrained model was selected (i.e., second model versus first model, and third model versus second model) if the following criteria suggested by Cheung and Rensvold [69] and Chen [70] were met: (a) the difference in RMSEA (ΔRMSEA) was lower than .01; (b) the differences in CFI (ΔCFI) and NNFI (ΔNNFI) were equal to or greater than -.01.

Table 2 shows the results of the measurement invariance testing. Metric and scalar invariance were supported across gender and samples because changes in RMSEA were lower than .01 and changes in CFI and NNFI indexes were equal to or greater than -.01 in the comparison of the models.

**Table 2:** Measurement invariance across clinical and nonclinical samples and gender.

Model	RMSEA	ΔRMSEA	CFI	ΔCFI	NNFI	ΔNNFI
Measurement invariance across sample						
MG Baseline model	.0749		.982		.977	
Metric invariance	.0763	-.0014	.979	-.003	.976	-.001
Scalar invariance	.0754	.0009	.977	-.002	.977	.001
Measurement invariance across gender						
MG Baseline model	.0854		.978		.972	
Metric invariance	.0850	.0004	.975	-.003	.972	.000
Scalar invariance	.0852	-.0002	.973	-.002	.972	.000

*Internal consistency*. The GTQ obtained alpha coefficients of .89 (95% CI [.88, .91] and .90 (95% CI [.87, .92]) and omega coefficients of .90 (95% CI [.88, .91]) and .90 (95% CI [.87, .92]) for Samples 2 and 3, respectively.

*Validity evidence based on relationships with other variables*. Pearson correlations between the one-factor model scores estimates of the GTQ and the other scales were calculated to analyze convergent construct validity. Table 3 shows that, as expected, the GTQ showed moderate negative correlations with the GPQ-9. Regarding ACT processes, the GTQ showed negative correlations with experiential avoidance (i.e., AAQ-II scores), cognitive fusion (i.e., CFQ), and obstruction in values (i.e., VQ-Obstruction), and positive correlation with progress in values (VQ-Progress). As expected, negative correlations were found between the GTQ and repetitive negative thinking (i.e., PTQ) and with emotional symptoms (i.e., DASS-21). Lastly, the GTQ strongly correlated with satisfaction with life (i.e., SWLS) and general self-efficacy (i.e., GSES). Table 3 also shows the correlation coefficients disattenuated of measurement error [71]. As expected, these correlations were stronger for almost all correlation pairs.

**Table 3:** Pearson correlations and disattenuated correlations between the one-factor model scores estimates of the gtq and other relevant self-report measures in samples 2 and 3.

Measures	S	*N*	*r* with GTQ	Disattenuated *r* with GTQ
GPQ-9	2	463	-.37***	-.41
	3	125	-.17	-.19
AAQ-II	2	463	-.52***	-.58
	3	125	-.33***	-.37
CFQ	3	125	-.16	-.18
VQ—Progress	3	125	.55***	.64
VQ—Obstruction	3	125	-.22**	-.27
PTQ	2	463	-.56***	-.60
	3	125	-.17	-.18
DASS–Total	2	463	-.49***	-.53
	3	125	-.18*	-.20
DASS–Depression	2	463	-.49***	-.54
	3	125	-.28**	-.31
DASS—Anxiety	2	463	-.43***	-.49
	3	125	-.03	-.03
DASS–Stress	2	463	-.45***	-.51
	3	125	-.16	-.18
SWLS	2	463	.53***	.59
GSES	2	463	.72***	.81

*p < .05

**p < .01, *p* < .001. AAQ-II = Acceptance and Action Questionnaire–II; CFQ = Cognitive Fusion Questionnaire; DASS-21 = Depression Anxiety and Stress Scale– 21; GPQ-9 = Generalized Pliance Questionnaire– 9; GSES = General Self-Efficacy Scale; SWLS = Satisfaction with Life Scale; VQ = Valuing Questionnaire.

*Group differences in GTQ scores*. Independent sample t-tests were conducted to explore if there were differences in GTQ across gender and nonclinical and clinical participants. The tests indicated that there were statistically significant differences in gender, with men obtaining higher scores than females in Sample 2 (men: *M* = 57.52, *SD* = 9.95; women: *M* = 54.79, *SD* = 10.15; *t*(441) = 2.61, *p* = .009). Likewise, there were statistically significant differences between clinical and nonclinical participants (clinical participants: *M* = 48.36, *SD* = 10.81; nonclinical participants: *M* = 55.79, *SD* = 10.17; *t*(586) = 7.15, p < .001).

## Study 3: Criterion validity based on correlations with executive function tests

This study aimed to analyze the criterion validity of the GTQ by analyzing its correlations with executive functions tests.

### Materials and methods

The procedures followed in the research reported in the manuscript were approved by the Bioethics Committee of Fundación Universitaria Konrad Lorenz. Written informed consent was obtained in all studies reported in the manuscript. The dataset used in this study can be obtained at https://osf.io/r2gb4/.

#### Participants

*Sample 4*. An a priori power analysis was conducted in G*Power [72] specifying 80% statistical power to detect a one-tailed, medium-size correlation of .25 at an alpha level of .05. The results showed that 97 participants were required. Accordingly, the sample used in this study consisted of 105 adult participants (64.8% females) with an age range from 18 to 38 years (*M* = 22.94, *SD* = 4.62).

#### Instruments

*Generalized Tracking Questionnaire (GTQ)*. As the original 11-item version of the GTQ demonstrated good psychometric properties and the expected one-factor structure, it was administered in this study as it was designed. The alpha coefficient of the GTQ in this study was .88, whereas the omega coefficient was .88 (95% CI [.84, .92]).

*Neuropsychological Battery of Executive Functions and Frontal Lobes– 2 (BANFE-2) [73].* The BANFE-2 is a battery of 14 tests that evaluate executive functions. In this study, we used the following tests: (a) Semantic Classification that evaluates the productivity of semantic groups and abstract ability; (b) Self-Directed Signaling that evaluates visuospatial working memory to signal in a self-directed way a series of figures; (c) Visuospatial Working Memory that evaluates the ability to retain and reproduce the visuospatial sequence of a series of figures; (d) Verbal Fluency that estimates the ability to produce in a limited time a series of verbs; (e) Alphabetic Ordering of Words that estimates the ability to manipulate and order the verbal information contained in the working memory; (f) Tower of Hanoi that evaluates skills on sequential planning; and (g) Stroop Test that evaluates inhibitory control.

#### Procedure

The sample was recruited through announcements in social media in which potential participants were invited to get involved in a study consisting of evaluating memory, attention and other cognitive skills. The interested participants who met the inclusion criteria were invited to an assessment session that took part in a Clinical Psychology laboratory of a Colombian university. During this assessment session, participants first responded to a sociodemographic form and a questionnaire package that included the GTQ on the platform www.typeform.com. Afterward, participants performed the executive functions tests that were administered by an experimenter.

### Results and conclusions

Correlations and *t*-tests were computed in SPSS 25^©^. As most of the variables of the BANFE-2 were in an ordinal scale, one-tailed Spearman correlations were performed to assess the association between the GTQ and all test excepting the Alphabetic Ordering of Words. In the latter test, we conducted a one-tailed independent samples t-test to evaluate the mean differences in GTQ scores of participants who responded correctly to each of the three tasks of the test vs. participants who failed the task. Cohen’s d was computed in the online calculator https://www.psychometrica.de/effect_size.html [74] and were interpreted following the guidelines suggested by Cohen [75] (small effect: 0.20 to 0.49; moderate effect: 0.50 to 0.79; and equal or higher than .80, strong effect). With respect to Spearman correlations, they were interpreted following Lenhard and Lenhard [73]: small effect: .10 to .20; medium effect: .21 to .36; and equal or higher than .37, strong effect.

Table 4 shows the Spearman correlations obtained between the GTQ and the executive functions tests. The GTQ showed positive, statistically significant correlations with productivity in the Semantic Classification test, the number of correct responses in the Self-Directed Signaling test, and the scores in the Visuospatial Working Memory and Verbal Fluency tests. Regarding the Tower of Hanoi test, the scores on the GTQ correlated negatively with the number of movements and time needed to finish the task. Lastly, the GTQ scores showed a negative, statistically significant correlation with interference in the Stroop Test. Overall, all statistically significant correlations were in the moderate range.

**Table 4:** Spearman correlations between the GTQ and executive functions tasks in sample 4.

	*rho* with GTQ
Semantic Classification–Productivity	.25**
Self-Directed Signaling–Corrects	.21*
Visuospatial Working Memory	.31**
Verbal Fluency	.21*
Hanoi Tower–Movements	-.17*
Hanoi Tower–Time	-.18*
Stroop Test	-.20*

*p < .05

**p < .01.

Regarding the Alphabetic Ordering of Words test, almost all participants (101) completed the first task correctly. In the second task, 74 participants (70.5%) responded correctly. There were statistically significant differences between completer and noncompleter participants on GTQ scores (completers: *M* = 58.09, *SD* = 8.08; noncompleters: *M* = 53.42, *SD* = 9.99; *t*(103) = -2.52, *p* = .007, *d* = 0.52), with a medium effect size favoring the completers. Lastly, there were no statistically significant differences in the third task between completers and noncompleters (*t*(103) = 1.17, *p* = .12).

## Discussion

The concept of rule-governed behavior was coined to provide functional analytic accounts of complex human behavior including problem-solving, executive functions, and psychopathology [1,5,7,12,28]. The functional classes of rule-following suggested by Zettle and Hayes [10] attempted to provide explanations, among others, of the phenomenon of insensitivity to contingencies induced by rule-governed behavior. Since then, the terms pliance and tracking have been popular in behavior analysis and contextual behavioral science, also been incorporated in RFT and ACT [17]. Despite this popularity, the experimental analysis of the types of rule-following has found significant difficulties [29]. This is not surprising given the fact that pliance and tracking are listener-oriented concepts, which implies that speakers cannot produce them in a reliable way [17].

Given the difficulty found in the experimental analysis of pliance and tracking, researchers have begun to follow the complementary strategy of developing self-reports that explore the individual’s learning history. The first step in this direction was to develop measures of generalized pliance (the GPQ and GPQ-C), which have shown good psychometric properties and criterion validity. Importantly, scores on these instruments have shown strong positive correlations with contingency-shifting tasks such as the WCST [33]. However, to our knowledge, there was no self-report dedicated to measuring the skill on deriving and following tracks, which we have suggested to call generalized tracking. Accordingly, this study aimed to develop and analyze the psychometric properties and validity of the GTQ–a new measure of generalized tracking.

In Study 1, an initial pool of items was generated on a group basis following a definition of generalized tracking. This pool was discussed and given to three experts in RFT and RGB. The final pool consisted of 11 items that constituted the GTQ. The GTQ was administered to a sample of 460 undergraduates who did not find problems in item understandability. The exploratory factor analysis revealed that the GTQ seemed to be a unidimensional measure with all items showing high factor loadings and corrected item-total correlations. In Study 2, the confirmatory factor analysis showed that the one-factor model of the GTQ obtained a good fit to the data. The GTQ showed excellent internal consistency and measurement invariance across gender and clinical and nonclinical samples. Also, the GTQ correlated with measures of generalized pliance, experiential avoidance, cognitive fusion, values, emotional symptoms, repetitive negative thinking, general self-efficacy, and life satisfaction. Lastly, in Study 3, the GTQ showed theoretically coherent correlations with executive functions tests measuring inhibitory control, working memory, planning, and verbal fluency and productivity.

These findings preliminary support the reliability of the GTQ in terms of internal consistency. Further studies should analyze reliability based on test-retest correlations. In this sense, test-retest correlations should be strong because the GTQ is a trait-type measure. Regarding internal validity, the GTQ has shown to be a unidimensional measure in both the exploratory and confirmatory factor analyses. Importantly, the finding of measurement invariance implies that scores on the GTQ can be comparable across gender and clinical and nonclinical participants. As expected, nonclinical participants showed higher scores than clinical participants in the GTQ, which highlights the adaptive role of generalized tracking.

The correlations of the GTQ with the GPQ (generalized pliance) were negative and in the moderated to strong size. At first sight, it might be surprising that the correlation between both patterns of rule-governed behavior was not larger because sometimes pliance and tracking are mistakenly thought as opposite types of rule-following. However, it should be taken into account that pliance is thought to be a condition for the development of tracking. Accordingly, measures of generalized pliance and tracking might even correlate positively in childhood. However, this correlation might turn into a negative one during adolescence and adulthood. To test this relevant hypothesis, the GTQ should be adapted to measure generalized tracking in children in a similar way the GPQ was adapted to compose the GPQ-C.

The remaining correlations shown by the GTQ with self-report instruments were also theoretically coherent. Specifically, consistent with the ACT model, the GTQ showed strong negative correlations with experiential avoidance and repetitive negative thinking in nonclinical participants (i.e., Sample 3). This seems logical because individuals displaying generalized tracking might contact the counterproductive consequences of engaging in experiential avoidance and repetitive negative thinking. Additionally, the thinking process of individuals showing generalized tracking might be more concrete and oriented to problem-solving as compared to worriers and ruminators who engage in more abstract repetitive thinking [76]. Accordingly, training worriers and ruminators in generalized tracking might be a way to reduce their tendency to engage in repetitive negative thinking. The GTQ also showed strong negative correlations with emotional symptoms, which seems coherent with the previously commented correlations. Contrarily, the GTQ showed strong positive correlations with progress in values, life satisfaction, and general self-efficacy. The very strong correlation between the GTQ and self-efficacy might be related to the efficacy of the thinking process displayed by individuals with generalized tracking, which might lead to perceive themselves as capable of coping with stressors. Lastly, the correlations found in the clinical sample (i.e., Sample 3) were considerably lower than in the nonclinical sample. This might due to the higher homogeneity of this sample and its relatively small size.

The GTQ showed significant correlations with a wide range of executive functions tests in Study 3. The size of the correlations was small to moderate, but all were theoretically coherent with the functional-analytic account of executive functions provided by Hayes et al. [12] in terms of flexibility in rule-governed behavior (rule-generation and rule-following). This finding shows the criterion validity of the GTQ and, in conjunction with the correlations found between the GPQ and the WCST, encourage further theoretical and empirical analyses of executive functions in terms of rule-governed behavior.

The development of general patterns of rule-governed behavior, such as the GPQ and GTQ, might support functional-analytic research on complex human behavior in several ways. Firstly, administering these instruments when conducting experimental research on rule-governed behavior might help to explain the variability of the results obtained. Secondly, the development and relationship between pliance and tracking might be analyzed across age. Thirdly, these instruments might be useful to conduct longitudinal studies analyzing the effect of the different patterns of rule-governed behavior on mental health and behavioral effectiveness. Lastly, they might be useful to analyze mediators or moderators of psychological interventions, especially in ACT.

Some limitations of this study are worth mentioning. Firstly, the samples used in this study had a larger percentage of women than men. Secondly, the clinical sample was relatively small and only included participants seeking treatment for emotional disorders. Thirdly, the psychometric properties of the GTQ have been analyzed only in Colombia. Further studies should examine the functioning of the GTQ in other Spanish-speaking countries and other languages. Fourthly, the GTQ was designed to measure generalized tracking as averaged across contexts. However, contextualized measures of tracking might be more relevant in specific situations. Fifthly, although the GTQ was designed to be administered in the general nonclinical and clinical adult population, the initial analysis of the psychometric properties and factor structure of the GTQ was conducted in a sample of undergraduates (i.e., Study 1). Sixthly, we have not tested measurement invariance of the mode administration of the GTQ (i.e., paper-and-pencil vs. online). Lastly, more extensive analyses of the relationship of generalized tracking with executive functions should be conducted. Specifically, the relationship between GTQ scores and cognitive flexibility tests such as the WCST should be analyzed.

In conclusion, this study has shown that the GTQ seems to be a sound measure of generalized tracking. Although self-report measures are known to have significant limitations, the GTQ might open new directions in the research on functional classes of rule-governed behavior across different domains including clinical psychology, educational psychology, and neuropsychology.

## Supporting information

S1 Table Spanish version of the GTQ. (DOCX)

S2 Table English version of the GTQ. (DOCX)
