# Supplementary material for: Development and initial validation of the Generalized Tracking Questionnaire
Source: PLoS One. 2020 Jun 11;15(6):e0234393. doi: 10.1371/journal.pone.0234393 (PMC7289427; doi:10.1371/journal.pone.0234393)
Supplement: S1 Table — (DOCX) [file pone.0234393.s001.docx]

**S1 Table. Spanish version of the GTQ.**

**GTQ**

Debajo encontrará una lista de afirmaciones. Por favor, puntúe en qué grado cada afirmación ES VERDAD PARA USTED haciendo un círculo en los números de al lado. Utilice la siguiente escala para hacer su elección.

| **1** | **2** | **3** | **4** | **5** | **6** | | | | | **7** | | | |
| --- | --- | --- | --- | --- | --- | --- | --- | --- | --- | --- | --- | --- | --- |
| **Nunca es verdad** | **Muy raramente es verdad** | **Raramente es verdad** | **A veces es verdad** | **Frecuentemente es verdad** | **Casi siempre es verdad** | | | | | **Siempre es verdad** | | | |
| 1. Cuando veo que algo no está funcionando, intento algo diferente. | | | | | | 1 | 2 | 3 | 4 | | 5 | 6 | 7 |
| 1. Disfruto descubriendo cómo funcionan las cosas y llegando a mis propias conclusiones. | | | | | | 1 | 2 | 3 | 4 | | 5 | 6 | 7 |
| 1. Me adapto fácilmente a los cambios. | | | | | | 1 | 2 | 3 | 4 | | 5 | 6 | 7 |
| 1. Tengo facilidad para encontrar soluciones novedosas a los problemas. | | | | | | 1 | 2 | 3 | 4 | | 5 | 6 | 7 |
| 1. Tomo decisiones basándome en mi experiencia y no en lo que los demás dicen. | | | | | | 1 | 2 | 3 | 4 | | 5 | 6 | 7 |
| 1. Me gusta probar distintas maneras de hacer las cosas para ver cuál es mejor. | | | | | | 1 | 2 | 3 | 4 | | 5 | 6 | 7 |
| 1. Soy bueno encontrando formas más efectivas de realizar tareas. | | | | | | 1 | 2 | 3 | 4 | | 5 | 6 | 7 |
| 1. Si noto que algo no funciona, cambio mi forma de actuar rápidamente. | | | | | | 1 | 2 | 3 | 4 | | 5 | 6 | 7 |
| 1. Aprendo de las consecuencias de mis acciones con facilidad. | | | | | | 1 | 2 | 3 | 4 | | 5 | 6 | 7 |
| 1. Cuando me doy cuenta de que estoy equivocado, cambio mi forma de pensar y actuar. | | | | | | 1 | 2 | 3 | 4 | | 5 | 6 | 7 |
| 1. Tomo decisiones basándome en los resultados que he obtenido anteriormente. | | | | | | 1 | 2 | 3 | 4 | | 5 | 6 | 7 |
