# Supplementary material for: Development and initial validation of the Generalized Tracking Questionnaire
Source: PLoS One. 2020 Jun 11;15(6):e0234393. doi: 10.1371/journal.pone.0234393 (PMC7289427; doi:10.1371/journal.pone.0234393)
Supplement: S2 Table — (DOCX) [file pone.0234393.s002.docx]

**S2 Table. English version of the GTQ.**

**GTQ**

Below you will find a list of statements. Please rate how true each statement is for you by circling a number next to it. Use the scale below to make your choice.

| **1** | **2** | **3** | **4** | **5** | **6** | | | | | **7** | | | |
| --- | --- | --- | --- | --- | --- | --- | --- | --- | --- | --- | --- | --- | --- |
| **never**  **true** | **very seldom true** | **seldom**  **true** | **sometimes**  **true** | **frequently**  **true** | **almost always true** | | | | | **always**  **true** | | | |
| 1. When I see that something is not working, I try something different. | | | | | | 1 | 2 | 3 | 4 | | 5 | 6 | 7 |
| 1. I enjoy finding out how things work and reaching my own conclusions. | | | | | | 1 | 2 | 3 | 4 | | 5 | 6 | 7 |
| 1. I adapt easily to changes. | | | | | | 1 | 2 | 3 | 4 | | 5 | 6 | 7 |
| 1. I am able to find novel solutions to problems. | | | | | | 1 | 2 | 3 | 4 | | 5 | 6 | 7 |
| 1. I make decisions based on my experience and not on what others say. | | | | | | 1 | 2 | 3 | 4 | | 5 | 6 | 7 |
| 1. I like to try different ways of doing things to see which is better. | | | | | | 1 | 2 | 3 | 4 | | 5 | 6 | 7 |
| 1. I'm good at finding more effective ways to perform tasks. | | | | | | 1 | 2 | 3 | 4 | | 5 | 6 | 7 |
| 1. If I notice that something is not working, I change my way of acting quickly. | | | | | | 1 | 2 | 3 | 4 | | 5 | 6 | 7 |
| 1. I learn from the consequences of my actions with ease. | | | | | | 1 | 2 | 3 | 4 | | 5 | 6 | 7 |
| 1. When I realize that I am wrong, I change my way of thinking and acting. | | | | | | 1 | 2 | 3 | 4 | | 5 | 6 | 7 |
| 1. I make decisions based on the results I have obtained previously. | | | | | | 1 | 2 | 3 | 4 | | 5 | 6 | 7 |
